# Possibility of venoarterial extracorporeal membranous oxygenator being a bridging therapy for hemodynamic deterioration of pulmonary tumor thrombotic microangiopathy prior to initiating chemotherapy: A case report

**DOI:** 10.1097/MD.0000000000012169

**Published:** 2018-09-14

**Authors:** Yoshiaki Iwashita, Takuya Hiramoto, Kei Suzuki, Ryotaro Hashizume, Kazuo Maruyama, Hiroshi Imai

**Affiliations:** a Emergency and Critical Care Center, Mie University Hospital; b Department of Anesthesiology and Critical Care Medicine, School of Medicine, Mie University; c Department of Pathology and Matrix Biology, Mie University Graduate School of Medicine, Tsu, Mie, Japan.

**Keywords:** ECMO, gastric cancer, PTTM, right heart failure

## Abstract

**Rationale:**

Pulmonary tumor thrombotic microangiopathy (PTTM) is a rare but lethal complication of carcinoma, defined as non-occlusive pulmonary tumor embolism complicated by fibrocellular intimal proliferation of the small pulmonary arteries, with eventual occlusion of the pulmonary arteries. Hemodynamic deterioration caused by this condition leads to high mortality.

**Patient concerns:**

A 46-year-old woman who had undergone radiation therapy for anaplastic oligoastrocytoma and who was taking temozolomide presented with cough and palpitations.

**Diagnoses:**

A 12-lead electrocardiogram showed sinus tachycardia and SIQIII TIII, with negative T in V1–3. Ultrasound cardiography showed a distended right ventricle. Enhanced chest computed tomography showed no significant thrombus in the major pulmonary artery. The patient's condition deteriorated the next morning, with her blood pressure decreasing to 40 mmHg and her SpO_2_ unmeasurable. She suffered cardiac arrest.

**Interventions:**

We initiated venoarterial extracorporeal membranous oxygenation (VA-ECMO) and her blood pressure increased to 80 mmHg. Her hemodynamic status stabilized and she was weaned off VA-ECMO on intensive care unit (ICU) day 3.

**Outcomes:**

Gastroesophageal endoscopy on ICU day 4 revealed gastric cancer (Borrman type IV), and she arrested again and died on ICU day 5. Autopsy confirmed gastric cancer and PTTM.

**Lessons:**

VA-ECMO rapidly stabilized the hemodynamic status of this patient with PTTM, and may thus be a possible bridging therapy for deterioration of PTTM prior to initiating imatinib.

## 1 Introduction

Pulmonary tumor thrombotic microangiopathy (PTTM) is defined as nonocclusive pulmonary tumor embolism complicated by fibrocellular intimal proliferation of small pulmonary arteries, eventually causing occlusion of the pulmonary arteries.^[1]^ The patient presents with pulmonary hypertension, rapidly progressing to hemodynamic deterioration. Platelet-derived growth factor (PDGF) is known a key factor regulating the proliferation,^[2–4]^ and use of the PDGF receptor-tyrosine kinase inhibitor imatinib was shown to improve short-term survival.^[4–6]^ However, it is still difficult for patients to survive hemodynamic deterioration. Here we describe a patient with PTTM who was treated successfully for hemodynamic deterioration by venoarterial extracorporeal membranous oxygenation (VA-ECMO).

## 2 Case presentation

**Figure 1:**
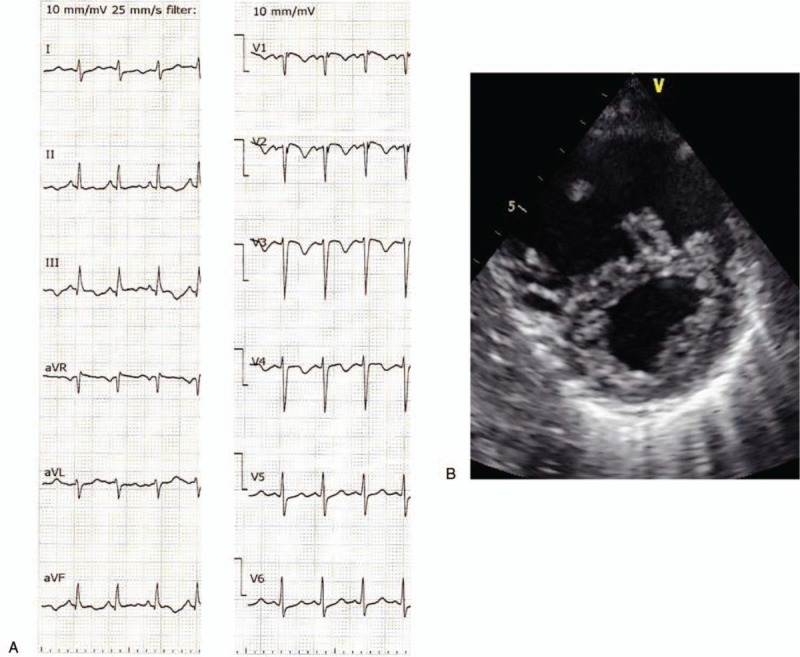
(A) Twelve-lead electrocardiogram on admission showed sinus tachycardia with SIQIII TIII, negative T in V1–3. (B) Short-axis ultrasound cardiogram showed left ventricle compressed by distended right ventricle.

**Figure 2:**
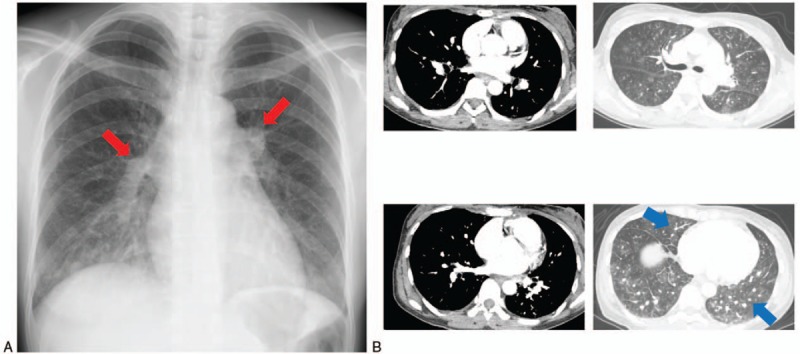
(A) Chest X-ray on admission showed distended pulmonary arteries (red arrowheads) and bilateral interstitial infiltrate. (B) Chest computed tomography on admission showed no pulmonary embolisms in major pulmonary arteries. Nodular opacities with tree-in-bud pattern are seen (blue arrowheads).

A 46-year-old woman who had received radiation therapy for anaplastic oligoastrocytoma and who was taking temozolomide presented to our hospital with cough and palpitations. Her vital signs on admission included blood pressure 115/83 mm Hg, heart rate 117 beats/min, body temperature 36.5°C, and SpO_2_ 96% (O_2_ 2 L/min cannula). A 12-lead electrocardiogram showed sinus tachycardia and SIQIII TIII, with negative T in V1–3 (Fig. 1A). An ultrasound cardiogram (UCG) showed a distended right ventricle, D-shape (+) (Fig. 1B), moderate tricuspid valve regurgitation, and moderate to severe pulmonary hypertension (tricuspid valve regurgitation pressure gradient max 59 mm Hg). Chest X-ray showed distended pulmonary arteries and interstitial lung infiltrate (Fig. 2A). Pulmonary embolism was suspected and the patient underwent emergency computed tomography (CT). However, enhanced chest CT showed no signs of thrombus in the major pulmonary arteries, but did show nodular opacities with tree-in-bud pattern (Fig. 2B). Abdominal CT showed a slightly distended gastric wall with some lymphadenopathies. Laboratory data showed the following: decreased platelets, 71 × 10^3^/μL; activated partial thromboplastin time, 30.7 seconds; prothrombin time-international normalized ratio, 1.19; fibrinogen, 100 mg/dL; and increased D-dimer, 20.08 μg/mL. No signs of infection were detected (white blood count 7450/μL, C-reactive protein 0.24 mg/dL, βd-glucan <2.4 pg/mL). The patient was hospitalized with suspected temozolomide-induced interstitial pneumonia complicated with disseminated intravascular coagulation and right heart failure. Steroid pulse therapy was initiated (methylprednisolone 1 g/d) for interstitial pneumonia, and recombinant thrombomodulin and continuous heparin infusion were administered for her hypercoagulable state.

The patient's condition deteriorated the following morning; her systolic blood pressure decreased to 40 mm Hg and her SpO_2_ was unmeasurable. She was moved to the intensive care unit (ICU) and intubated, and catecholamine administration was initiated. Her systolic blood pressure was 40 mm Hg and her arterial lactate level increased to 16.1 mg/dL on a regime of noradrenaline 0.3 μg/kg per min, dopamine 10 μg/kg per min, dobutamine 10 μg/kg per min, and vasopressin 2 U/h. However, UCG showed that the D-shape of her heart had worsened, and the patient suffered cardiac arrest. Although we suspected PTTM because of the rapid deterioration of her right heart failure, no definite diagnosis was made at this point, and VA-ECMO rescue therapy was initiated. The VA-ECMO conditions included a 22-Fr drainage catheter from the right femoral vein to the right atrium, and a 20-Fr arterial catheter for the left femoral artery. The pump was started as 2400 rpm and the resulting blood flow was about 3 L/min. After starting VA-ECMO, the patient's blood pressure increased to 80 mm Hg and her lactate level decreased.

**Figure 3:**
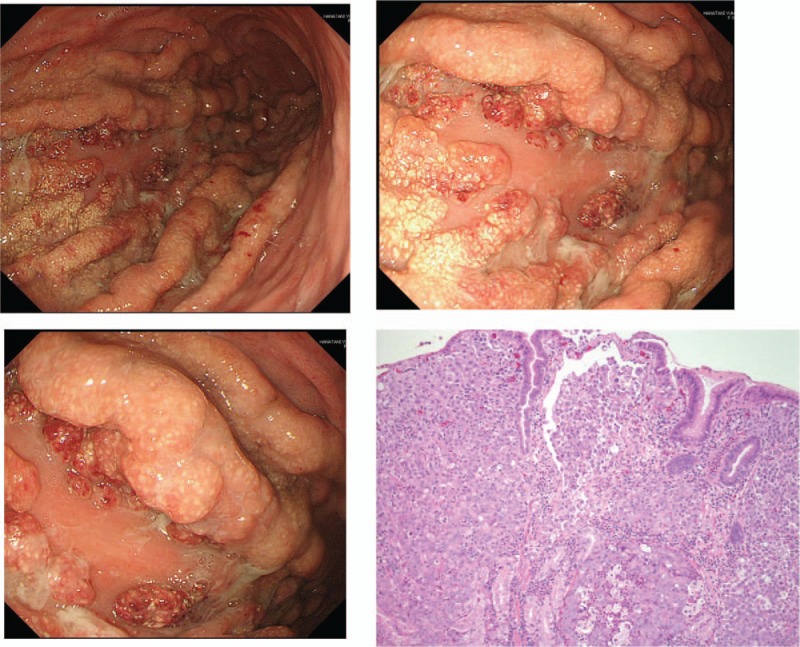
Gastroendoscopy showed Borrman type 4 tumor in the greater curvature. Histopathology showed a poorly differentiated adenocarcinoma with signet ring cells.

The patient's hemodynamic status stabilized on VA-ECMO. She started to be weaned off VA-ECMO and catecholamines the following day, and VA-ECMO was ceased on ICU day 3. The patient underwent gastroesophageal endoscopy on ICU day 4 to screen for possible gastric cancer, as a potential cause of her PTTM, and a gastric tumor (Borrman type IV) was detected (Fig. 3). Biopsy showed a poorly differentiated adenocarcinoma with signet ring cells. Her carcinoembryonic antigen (CEA) level was 9.1 ng/mL and CA-19-9 was 44,793 U/mL. The patient suddenly arrested again on ICU day 5 and could not be resuscitated.

**Figure 4:**
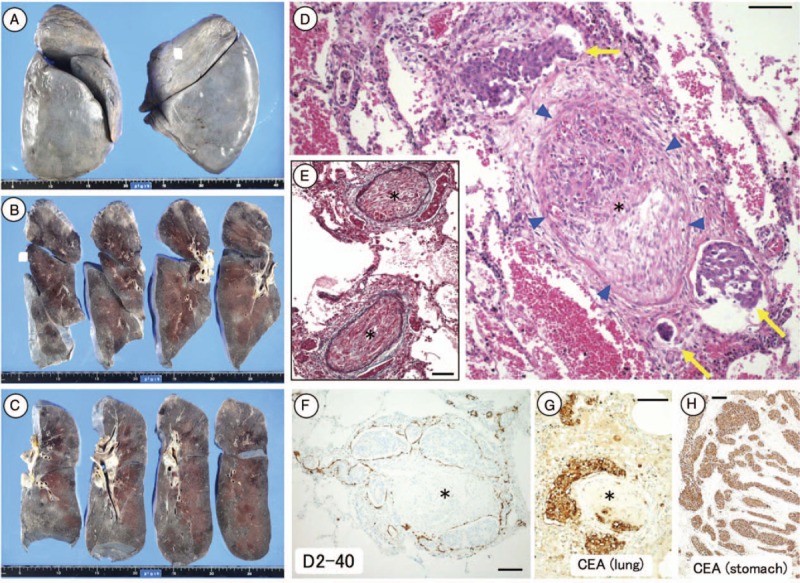
Macroscopic and microscopic findings at autopsy. No macroscopic nodular lesions were evident in either lung, except for bilateral hilar lymph node metastases (A–C). Distinct fibrocellular stenosis of the pulmonary arterioles was detected histologically (D) (arrowhead). Tumor cells were either detectable or not present within the vessel. Elastica van Gieson staining attributed the fibrocellular stenosis to intimal proliferation (E). Periarterial lymphatics filled with tumor cells (i.e., lymphangiosis carcinomatosa) (D–F, arrow). The lung-infiltrating tumor cells and primary tumor cells in the stomach were both carcinoembryonic antigen-positive (G, H), indicating that the tumor cells in the lung originated from the stomach. Scale bars in D–H: 100 μm. Asterisks in D–G: severe stenotic or occluded pulmonary arterioles.

Autopsy revealed a gastric tumor in the greater curvature of the stomach and distended bilateral hilar lymph nodes. Microscopic examination of the gastric tumor showed a poorly differentiated adenocarcinoma, and blood vessels filled with the adenocarcinoma. Anti-CEA immunostaining was positive for tumor cells. Although no lung nodular lesions were detected macroscopically (Fig. 4A–C), histological examination revealed fibrocellular stenosis of the pulmonary arterioles (Fig. 4D) and fibrocellular intimal proliferation in the pulmonary vessels (Fig. 4E and F). The lung-infiltrating tumor cells were positive for CEA by immunostaining (Fig. 4G and H). These findings confirmed a diagnosis of PTTM caused by gastric cancer. Blood vessels in other organs, including the liver, pancreas, uterus, and ovaries, were also filled with tumor cells and showed intimal proliferation.

## 3 Discussion and conclusion

We report a patient with a history of astrocytoma who presented with right heart failure and interstitial lung infiltrate, rapidly progressing to hemodynamic deterioration. The patient was treated successfully with VA-ECMO but subsequent gastroendoscopy revealed gastric cancer. The patient unfortunately deteriorated again before a pathological diagnosis could be made, and we were therefore unable to treat her with imatinib; however, this experience suggests that VA-ECMO may provide effective rescue therapy for patients with rapid deterioration of PTTM.

The clinical characteristics of PTTM include rapid progression of respiratory insufficiency accompanied by a hypercoagulable state, without embolism in the major pulmonary arteries.^[7]^ Although the rapid worsening of the manifestations make an antemortem diagnosis of PTTM difficult,^[8–11]^ we suspected PTTM at an early stage because of the signs of right heart failure on electrocardiography and UCG, with no sign of pulmonary embolism on enhanced CT. We could not find any previous report suggesting astrocytoma as a cause of PTTM and we therefore sought to identify other possible causes of the patient's PTTM. This early suspicion of adenocarcinoma led to the confirmation of gastric cancer by endoscopy as soon as the patient was weaned off VA-ECMO.

The tyrosine kinase inhibitor imatinib is a molecular targeting drug that inhibits the PDGF receptor. Limited reports have suggested that imatinib may be effective in patients with PTTM.^[4–6]^ Minatsuki et al reported a patient with PTTM and gastric cancer who was treated with imatinib, total gastrectomy, and adjuvant chemotherapy, who survived for at least 1 year. They also reported that histological examination of biopsies taken before and after imatinib therapy showed revascularization of the thrombotic vasculature.^[4]^ However, although imatinib therapy can recanalize the fibrocellular intimal proliferation, it is not effective for rapid hemodynamic deterioration. Furthermore, it is also difficult to start chemotherapy before a definite diagnosis has been made.

VA-ECMO is increasingly used to treat the most severe form of pulmonary embolism. VA-ECMO drains blood from the right atrium and provides retrograde systemic circulation from the femoral artery, allowing it to bypass the pulmonary circulation. VA-ECMO is thus an effective option for patients with severe right heart failure. A recent Japanese nationwide database study revealed an in-hospital mortality among pulmonary embolism patients who required VA-ECMO of 64% (226/353 cases). Although this mortality is still high, it is still lower than overall VA-ECMO in hospital mortality for out of hospital cardiac arrest (72.5%).^[12]^ The risks for introducing ECMO includes further coagulopathy due to start of heparin infusion, limb ischemia and infection, etc. However, the benefit of ECMO is a resuscitation. Thus the risks can be exceeded by the benefit, only when the patient is in a life-threatening or cardiac arrest situation. These results suggest that VA-ECMO may offer a temporary rescue option for PTTM patients.

The current patient's hemodynamic status was successfully stabilized by VA-ECMO, but they were then weaned off ECMO without starting imatinib therapy and their condition subsequently deteriorated. Thus the main limitation of this case report was that we are not sure that we could save this patient if we started imatinib on ECMO. However, considering 2 previous case reports, describing PTTM patients whom hemodynamic deterioration was treated successfully by VA-ECMO and started imatinib or chemotherapy during VA-ECMO run and achieved short-term survival.^[3,13]^ These previous cases and our current experience suggest that the appropriate treatment strategy for PTTM should include VA-ECMO for hemodynamic deterioration, with the early use of imatinib for recanalization of pulmonary thrombosis by fibrocellular intimal proliferation and chemotherapy for the causative cancer initiated during the VA-ECMO run. In conclusion, VA-ECMO should be considered as a possible bridging therapy for patients with undiagnosed deterioration of PTTM.

## Acknowledgments

The authors thank Drs A. Ito, E. Kawamoto, K. Yokoyama, Y. Omori, M. Fujioka, and T. Takeda, members of the Emergency and Critical Care Center, S. Ishiyama, A. Matsuda, Department of Cardiology, K. Asayama, Y. Takahashi, Department of Pulmonology, T. Matsubara, Department of Neurosurgery, Mie University Hospital, for their contributions. The authors also thank Susan Furness, PhD, from Edanz Group (www.edanzediting.com/ac) for editing a draft of this manuscript.

## Author contributions

**Supervision:** Kazuo Maruyama, Hiroshi Imai.

**Writing – original draft:** Yoshiaki Iwashita.

**Writing – pathology part:** Ryotaro Hashizume.

**Writing – review & editing:** Takuya Hiramoto, Kei Suzuki, Ryotaro Hashizume.

Yoshiaki Iwashita orcid: 0000-0002-7054-8448
